# Snacking Behaviors in Relation to Stress and Physical Activity Using Automated Dietary Assessment: Observational Study

**DOI:** 10.2196/77046

**Published:** 2026-07-23

**Authors:** Femke J de Gooijer, Alex van Kraaij, Marlou Lasschuijt, Sander Hermsen, Edith J M Feskens, Guido Camps

**Affiliations:** 1 Department of Agrotechnology and Food Sciences Division of Human Nutrition and Health Wageningen University & Research Wageningen The Netherlands; 2 OnePlanet Research Center Wageningen The Netherlands; 3 Prevention Hub Radboud University Medical Center Nijmegen The Netherlands

**Keywords:** snacking, stress, physical activity, sensor technology, ecological momentary assessment, wearables

## Abstract

**Background:**

Snacks contribute 19% to 34% of daily energy intake, yet studying snacking behaviors faces challenges due to self-report biases. The SnackBox, an automated dietary assessment tool, enables structured measurement of snacking in seminaturalistic settings.

**Objective:**

This study explored the relationships between snacking and perceived stress (case study 1) and physical activity (case study 2) using the SnackBox technology.

**Methods:**

Forty-seven office workers (n=29, 61.7% female; mean age 28.8, SD 4.1 years) used the SnackBox at work and home for 2 weeks. The device recorded snack consumption (grams and time). Participants reported perceived stress via ecological momentary assessments and wore a Chill+ band and a Garmin wearable to track physical activity.

**Results:**

Across the study period, 1455 ecological momentary assessments, 2933 hours of wearable data, and 2199 snacking events were recorded. Participants consumed a daily average of 769 (SD 772) kcal from snacks. Stress did not significantly impact snacking likelihood (*χ*^2^_1_=0.2; *P*=.69). Snacking was more likely before vigorous physical activity (odds ratio 2.00; SE 0.39; *P*<.001).

**Conclusions:**

The SnackBox demonstrated utility as a technology probe for exploring relationships between snacking and physiological and contextual factors. This approach enhances our understanding of snacking and supports personalized interventions and predictive models.

**Trial Registration:**

ISRCTN Registry ISRCTN45046481; https://www.isrctn.com/ISRCTN45046481

## Introduction

Snacks—foods and beverages consumed between meals—are a significant part of our modern diets. In the Netherlands, 28% of daily energy intake occurs outside breakfast, lunch, and dinner [1], and similar percentages are reported in other countries, such as the United States (23%) [2], Canada (24%) [3], Greece (34%) [4], France (19%) [5], and Brazil (21%) [6]. While the relationship between snacking and BMI remains inconclusive [7-9], individuals with overweight or obesity tend to consume more energy-dense snacks high in sugar and fat, and their snack consumption is positively associated with adiposity [10]. In the Netherlands, snacks also contribute significantly to sugar intake, accounting for half of daily mono- and disaccharide consumption [1].

Understanding snacking behaviors, snack choices, and their underlying factors is essential for improving diet quality [7,11]. Various factors influence snacking, including culture [12], social and physical environment [13,14], hunger [15], physical activity (PA) [16], sleep [17], distraction [18], and affect [19]. However, research into snacking behavior has primarily relied on 2 distinct methodologies: self-reported assessments and controlled laboratory experiments. Each approach has advantages and limitations. Self-reports enable assessment in naturalistic environments; nevertheless, their completion is perceived as burdensome [20] and is susceptible to inaccuracies from both intentional and unintentional response biases, as well as challenges in estimating portion sizes. Underestimation of self-reported energy intake can reach 47% [21], and snack consumption, in particular, is prone to being underreported [22,23]. In contrast, controlled laboratory environments facilitate precise monitoring of food intake but are less suitable for prolonged data acquisition. Furthermore, artificial laboratory environments may not reflect real-life eating behaviors. For instance, the impact of portion size on intake is less pronounced in the laboratory compared with at home [24], different physiological responses are observed [25], eating rates might differ [25,26] and real-life stressors are known to exert a more pronounced impact than artificial stressors [27].

To address these challenges, we developed the SnackBox, an automated dietary assessment device designed to enable structured measurement of snacking behavior in seminaturalistic settings [28]. The SnackBox is a rectangular platform containing electronics with 3 sensing coasters, each storing a snack container or beverage bottle, and automatically recording the amount consumed (in grams) and the time of consumption. A prior validation study demonstrated that the SnackBox estimated portion sizes with an intraclass correlation coefficient of 0.80 and a mean percentage error of −6%, outperforming self-report methods, which yielded an intraclass correlation coefficient of 0.60 and a mean percentage error of −40% [28]. Detection of snack events showed 81% agreement with an observational reference, compared with 54% for self-report [28]. The device operates with minimal researcher or user intervention, and gathered data are processed in real time, enabling the triggering of just-in-time adaptive interventions or ecological momentary assessment (EMA) surveys upon snack event detection to capture, for example, eating motives or contextual factors.

Understanding how transient psychological and physiological states shape snacking in daily life remains an open question that is difficult to address with either self-report methods or controlled laboratory studies. In case study 1, we investigated the effect of self-administered stress (measured using EMA) on snacking behaviors. While research on the impact of stress—whether induced or perceived—on snacking is mixed [29,30], a meta-analysis by Hill et al [19] found a small but significant positive relationship between stress and food intake. They also identified restrained eating traits as a moderating factor in the link between stress and unhealthy eating, with other studies highlighting emotional eating traits [31] and sex [32] as moderators. In case study 2, we examined snacking in relation to PA. Oh and Taylor [33] examined the acute effects of exercise on snack cravings and self-regulation and found that both moderate and vigorous exercise reduced snack cravings and attentional bias toward food cues; however, naturalistic data linking objectively measured and continuously monitored PA to snacking behavior in free-living settings remain scarce [9,11]. At the physiological level, exercise temporarily reduces subjective feelings of appetite during and for 30 to 60 minutes after activity, a phenomenon termed exercise-induced anorexia [34], though these acute effects do not appear to translate into reductions in overall energy or macronutrient intake.

The aim of this study was therefore to explore the relationships between snacking and perceived stress (case study 1) and PA (case study 2), using the SnackBox to link precise, time-stamped intake data [28] with concurrent physiological and contextual measures in seminaturalistic settings.

## Methods

### Study Design

We conducted a secondary data analysis of data collected to validate the SnackBox [28]. In this study, participants used the SnackBox to assess their snacking behavior for 5 days. Simultaneously, participants completed EMA questionnaires to assess their mood states (case study 1) and wore activity trackers (case study 2) to record physiological data.

### Ethical Considerations

This study was preregistered at the ISRCTN Registry (ISRCTN45046481) and was deemed exempt from review for ethics approval according to the Dutch Medical Research Involving Human Subjects Act (WMO) by the medical-ethical committee of the Máxima Medical Center in Veldhoven, the Netherlands (N21.099). An extensive risk assessment did not reveal any risks exceeding acceptable limits, while possible risks were mitigated as much as possible. This study adhered to the 2019 amendment of the Declaration of Helsinki. Prior to the study period, all participants gave informed consent.

### Recruitment

We recruited 47 healthy adults aged 18 to 49 years through a recruitment strategy involving poster advertisements and flyer distribution across the campuses of Wageningen University and Radboud University Nijmegen. Inclusion criteria were having a sedentary desk job (to minimize study material transport), being in good general health (self-reported), and having no dietary restrictions that could interfere with the study, as the fixed snack selection could not adequately accommodate participants with substantial dietary or allergy-related requirements.

### General Procedures

On day 0, participants visited the research facility for an intake session. We informed them about the study protocols, and participants installed the necessary apps (ImecQ and Traqq) on their mobile phones. A suitable mobile phone (Android) was provided when needed. Participants also received 2 activity trackers (Chill+ [Imec] and Vivosmart 4 [Garmin]) and selected 2 beverages and 3 snacks from a standardized offering of 9 beverages and 15 snacks (Multimedia Appendix 1), chosen to include both sweet and salty options and to vary in accordance with the Dutch dietary guidelines, while ensuring adequate shelf life across the study period. Selected items were provided ad libitum. Participants then scheduled 5 business days, not necessarily consecutive, within a 2-week timeframe for the recording of snacking behaviors in both work and home settings.

After the intake session, participants completed an intake questionnaire with questions on general demographics together with the Dutch Eating Behavior Questionnaire (DEBQ) [35], the Depression Anxiety Stress Scale (DASS) [36], and the Perceived Stress Scale (PSS) [37].

During these measurement days (days 1-5), participants were explicitly instructed to consume only the food and beverages that they selected during the intake session between their regular meals and to consume these foods only from the SnackBox. Exceptions were made for water, tea, and coffee without additives. Throughout the measurement day, participants wore the 2 activity trackers from wake-up until bedtime, received prompts to complete EMA questionnaires in the ImecQ app, and were reminded 4 times to log their main meals in the Traqq app.

On day 6, participants returned to the research facility to hand over all study equipment, and we asked them to complete an evaluation questionnaire. Participants received a €40 (€1=US $ 1.14 as of June 23, 2026) voucher for a Dutch webstore (bol.com). Data collection was conducted between May and August 2022.

### Assessment of Snacking Behavior

**Figure 1:**
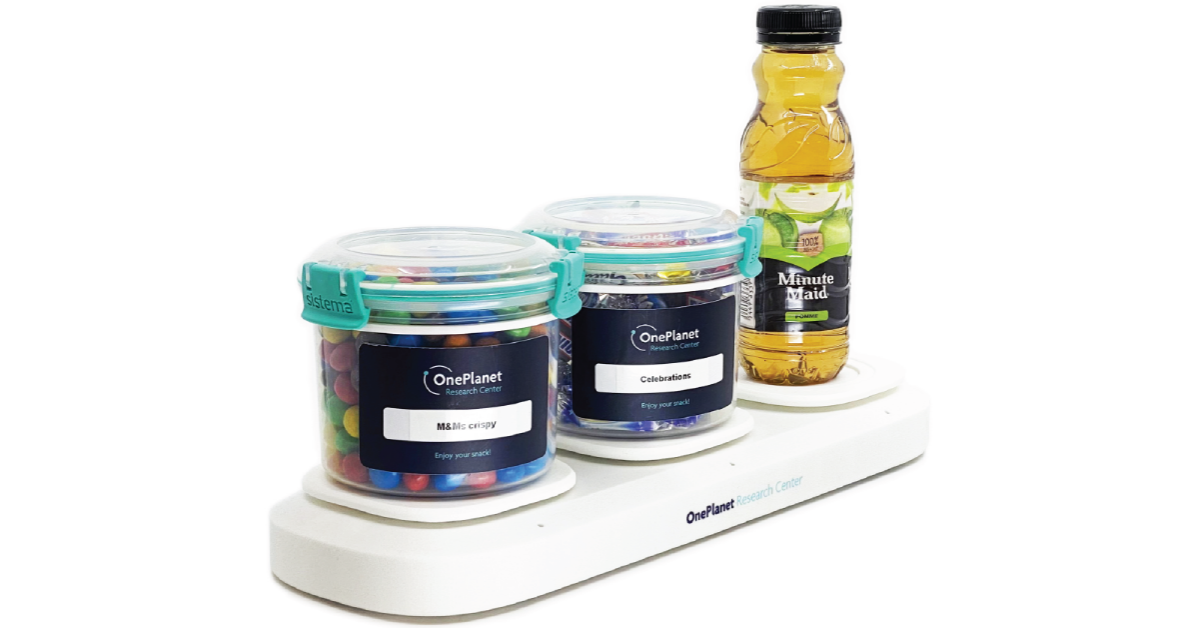
The SnackBox with 3 weighing stations for storing snack containers and beverage bottles. Estimates of consumed quantities and the type of snack or beverage are automatically recorded.

The SnackBox (Figure 1) is a rectangular platform (15×30 cm) with 3 coasters, or weighing stations, each designed to hold a snack container (Ø 10 ×10 cm) or beverage bottle (up to 600 g). Weight differences recorded at each station are used to estimate the amount consumed over time, and radio-frequency identification (RFID) labels identify the type of snack or beverage. Filtering of weight data, measurement precision, and validity under seminaturalistic conditions have been established in prior work [28]. Given its form factor, the device is suited to fixed desk-based or home settings rather than fully portable real-world use.

We directed participants to keep the SnackBox on their desk during work hours and in their living room during leisure time, while always having 2 snack containers and one beverage on the 3 coasters and to refill snack containers when empty. Participants were instructed to consume at least one bite of a snack or sip of a beverage per measurement day to confirm device functionality, as the dataset was originally collected to validate the SnackBox [28]. Furthermore, we asked participants to exclusively consume food between meals from the SnackBox and only when the device was turned on. If participants were unable to complete a full 5 measurement days due to illness or technical issues, the remaining data points were still included for subsequent analysis. Consequently, each participant provided a dataset encompassing 4 to 5 days, which included information about the types of snacks and beverages consumed, along with their estimated consumption (in grams) and corresponding timestamps.

### Assessment of Meals

We utilized the Traqq app [38] to collect self-reported data on the consumption of main meals, water, tea, and coffee. Designed to mitigate memory bias, Traqq permits shorter recall periods compared with standard 24-hour recalls. Participants were introduced to the app during the intake session. On each measurement day, participants received 3 prompts (at 11:30 AM, 4:30 PM, and 9:30 PM) to record their intake over the preceding 5 hours. At 7:30 AM, participants received an additional prompt to record their intake over the past night. A dietitian computed the nutritional values of registered items, and household measures were converted into grams.

### Data Analysis

All data analyses were conducted in R (version 4.2.1; R Foundation for Statistical Computing) [39]. Basic statistics were calculated for parameters from the intake questionnaire and for daily snack, drink, and meal consumption. The temporal distribution of eating events across the day was visualized using kernel density plots (*ggplot2* package [40]). One participant did not complete the intake questionnaire, and these data were excluded from analysis where sample characteristics (eg, sex and behavioral eating traits) were involved. All mixed models used in the 2 case studies were estimated using the “lmer” function, and the significance of fixed effects was assessed using likelihood ratio tests by comparing nested models using the “anova” function (*lme4* package [41]). Post hoc comparisons were done using the *emmeans* package [42].

### Case Study 1: Stress and Snacking

#### Procedure

Throughout the measurement days, participants received EMA questionnaire invitations via the ImecQ app on their phones to record self-reported mood states. The EMA questionnaire consisted of 8 questions: 2 questions for validation purposes, followed by 5 questions to assess different mood states (eg, “Right now, I feel Relaxed/Stressed”) on a visual analog scale (VAS) ranging from 0 (relaxed) to 100 (stressed). Invites were automatically sent at startup and shutdown of the SnackBox, randomly within a 1-hour time frame around 4 moments throughout the day (11 AM, 2 PM, 4 PM, and 9 PM), and 10 minutes after the detection of a snack event by the SnackBox. A maximum of one EMA prompt per 30 minutes was set. Both the ImecQ app and the SnackBox logged data to the same online backend, such that their timestamps were inherently synchronized.

#### Data Analysis

**Figure 2:**
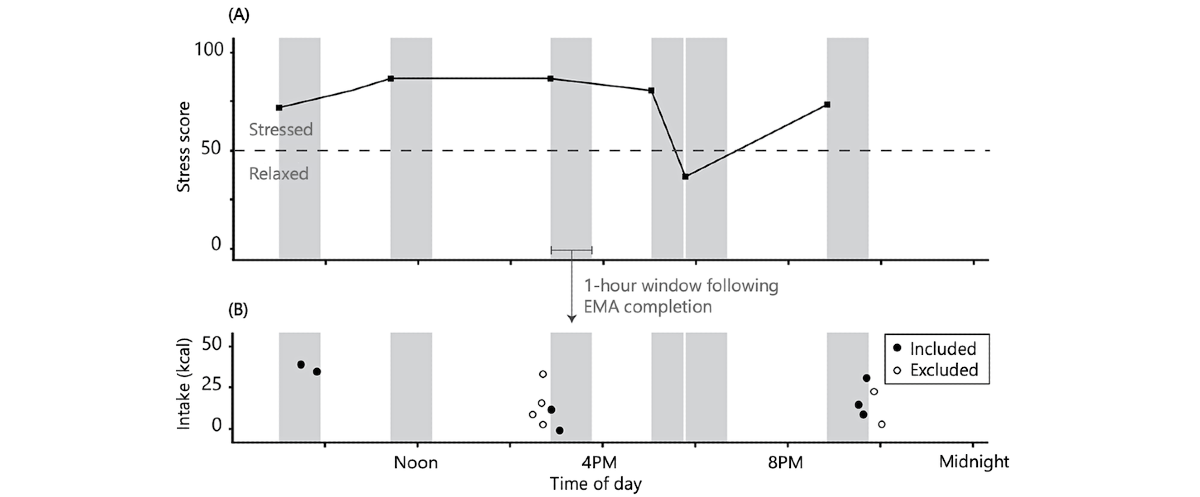
Example data illustrating the integration of stress measures and snacking data. (A) Ecological momentary assessment (EMA)–obtained self-administered stress levels (visual analog scale) throughout the day, categorized as stressed (score>50) or relaxed (score<50). (B) Corresponding SnackBox-recorded snack intake. Shaded areas indicate 1-hour windows after a completed EMA from which snack data were included in the analysis.

To examine the relationship between EMA-reported stress and snack consumption within the following hour, we defined time windows starting from the EMA response time and lasting either 1 hour or until the next EMA, whichever occurred first (Figure 2). Snack consumption was summarized as the occurrence of snacking in this window (ie, yes or no) and as the total caloric intake per window. For the VAS scores, responses exactly at 50 were excluded, as these indicated no adjustment to the slider. Scores below 50 were categorized as “relaxed,” and scores above 50 as “stressed.”

A mixed-effects logistic regression was used to assess whether the level of stress (ie, relaxed or stressed) predicted snack occurrence (ie, yes or no), while controlling for the time of day. Participants were included as a random intercept to account for individual variation. To assess the moderating effects of sex, emotional eating (DEBQ–emotional eating [DEBQ-E]), and restrained eating (DEBQ-R) scores, interactions (stress×sex, stress×DEBQ-E, and stress×DEBQ–restrained eating [DEBQ-R]) were added individually to the model. Subsequently, we repeated this process for caloric intake from snacks as the outcome variable, using a linear regression model that included only instances where snacking occurred.

### Case Study 2: PA and Snacking

#### Procedure

On each measurement day, from wake-up until bedtime, participants wore 2 wearable sensors to obtain physiological measures: a Chill+ and a Vivosmart 4. The Chill+ band gathered data on photoplethysmography, galvanic skin response, skin temperature, and acceleration. The Garmin band was used to obtain heart rate estimations based on photoplethysmography data. For this case study, acceleration data from the Chill+ band were used. Prior to each study period, the Chill+ band was synchronized to the SnackBox timestamps.

#### Data Analysis

To quantify PA, the metabolic equivalent of tasks (METs) was determined from accelerometer data. METs indicates energy expenditure as a multiple of resting metabolic rate [43]. This was done using an internally developed and trained machine learning–based model that used 5 minutes of raw accelerometer data as input and provided a METs value for every 5-minute window as output. METs were categorized as limited PA (METs<3), moderate PA (3≤METS≤6), or vigorous PA (METS>6) according to William et al [43]. Daily PA was summarized as the sum of minutes spent in vigorous PA and as the average METs per day. Given evidence that exercise-induced effects on appetite and snack intake are most pronounced after vigorous intensity exercise [44], only vigorous PA was included in analyses examining the relationship between daily PA and snack consumption.

The relationship between daily snack intake and daily PA was first studied using 2 mixed-effects models. The sum of calories consumed from snacks over the day was taken as the outcome variable, and the sum of minutes spent in vigorous PA and the number of days into the study were included as fixed effects. Participants were included as a random intercept to account for individual variation. This analysis was repeated using the average METs as the main effect.

**Figure 3:**
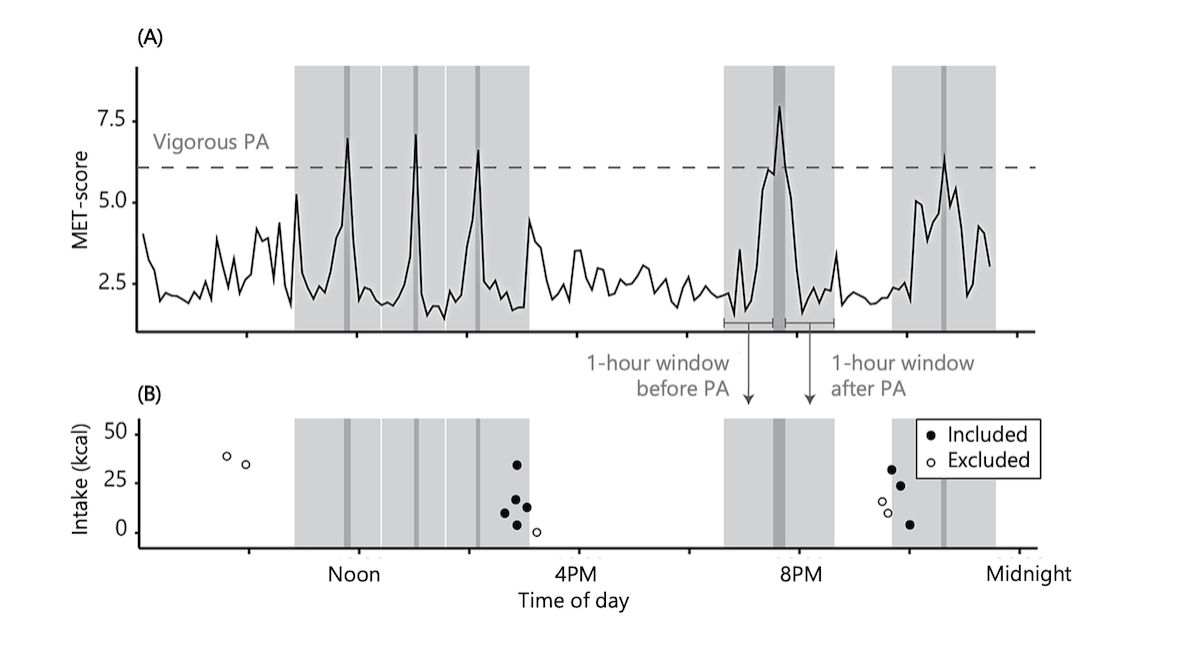
Example data illustrating the integration of physical activity and snacking data. (A) Wearable-obtained physical activity intensity (metabolic equivalent of task [METs]) throughout the day, with the vigorous activity threshold (METs>6) shown by a dashed line. (B) Corresponding SnackBox-recorded snack intake that was included in or excluded from the analysis. Shaded areas indicate 1-hour windows before and after vigorous activity from which snack data were included in the analysis, with overlapping periods assigned to the nearest vigorous activity event. PA: physical activity.

Second, 1-hour windows before and after periods of vigorous PA were identified (Figure 3). Overlapping hours, where time fell both within the hour after and before vigorous activity, were assigned to the period of vigorous PA to which they were closest. For instance, if vigorous PA was observed from 11:50 to 11:55 and from 12:50 to 12:55, then 11:55 to 12:22 would be labeled “after PA,” while 12:23 to 12:50 would be labeled “prior to PA.” Snack consumption in these windows was summarized as the occurrence of snacking (ie, yes or no) and as the total caloric intake per window. A mixed-effects logistic regression was used to assess whether time relative to vigorous PA (ie, prior to or after) predicted snack occurrence (ie, yes or no), while controlling for the time of day. Participants were included as a random intercept to account for individual variation. Subsequently, this process was repeated for caloric intake from snacks as the outcome variable, using a linear regression model that included only instances where snacking occurred.

## Results

### Sample Characteristics

**Figure 4:**
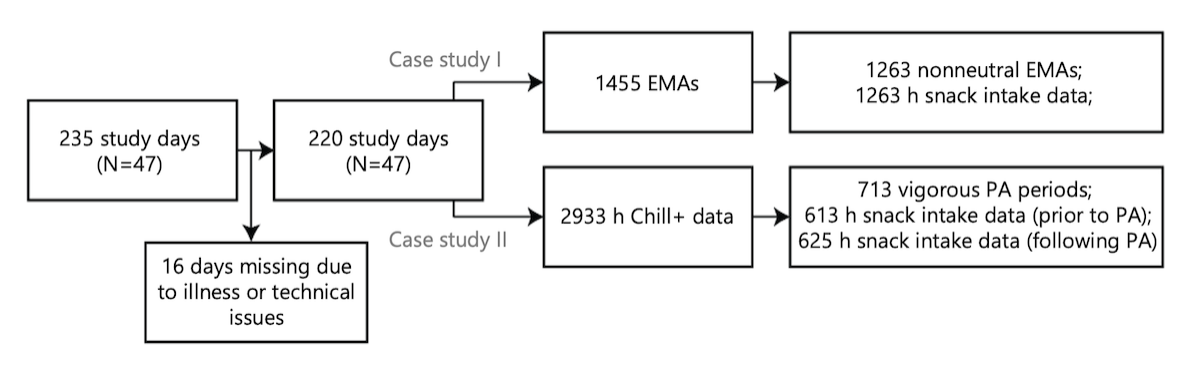
Flowchart of data included in both case studies. EMA: ecological momentary assessment; PA: physical activity.

The final sample consisted of 47 adults (n=29, 61.7% female; n=17, 36.2% male; and n=1, 2.1% missing data) with a mean age of 28.8 (SD 4.4) years (Table 1). A flowchart of included data for both case studies is presented in Figure 4. Our sample scored similarly on external eating but lower on emotional and restrained eating compared with the general population [45]. Mean DASS scores for depression, anxiety, and stress were in the normal range [36], and mean PSS scores indicated moderate perceived stress [37].

**Table 1:** General characteristics of the study population (N=47).

Characteristics			Values
Age (years), mean (SD)			28.8 (4.4)
**Sex, n (%)**			
	Female	29 (61.7)	
	Male	17 (36.2)	
	Unknown^a^	1 (2.1)	
**Dutch Eating Behavior Questionnaire, mean (SD)^b^**			
	External eating	2.86 (0.75)	
	Emotional eating	1.76 (1.21)	
	Restrained eating	1.82 (0.81)	
**Depression Anxiety and Stress Scale Questionnaire, mean (SD)^c^**			
	Depression	2.89 (2.56)	
	Anxiety	2.70 (2.28)	
	Stress	5.46 (3.17)	
Perceived Stress Scale, mean (SD)^d^			15.6 (5.79)

^a^One participant did not complete the intake questionnaire.

^b^Dutch Eating Behavior Questionnaire scores range from 1 to 5. General Dutch population scores are mean 2.82 (SD 0.56) for external eating, mean 2.43 (SD 0.81) for emotional eating, and mean 2.66 (SD 0.77) for restrained eating [45].

^c^Depression Anxiety and Stress Scale Questionnaire scores in the range of 0-9 (depression), 0-7 (anxiety), and 0-17 (stress) are interpreted as normal [36].

^d^Perceived Stress Scale: 0-13 indicates low, 14-26 moderate, and 27-40 high stress [37].

### Snack and Meal Consumption

**Figure 5:**
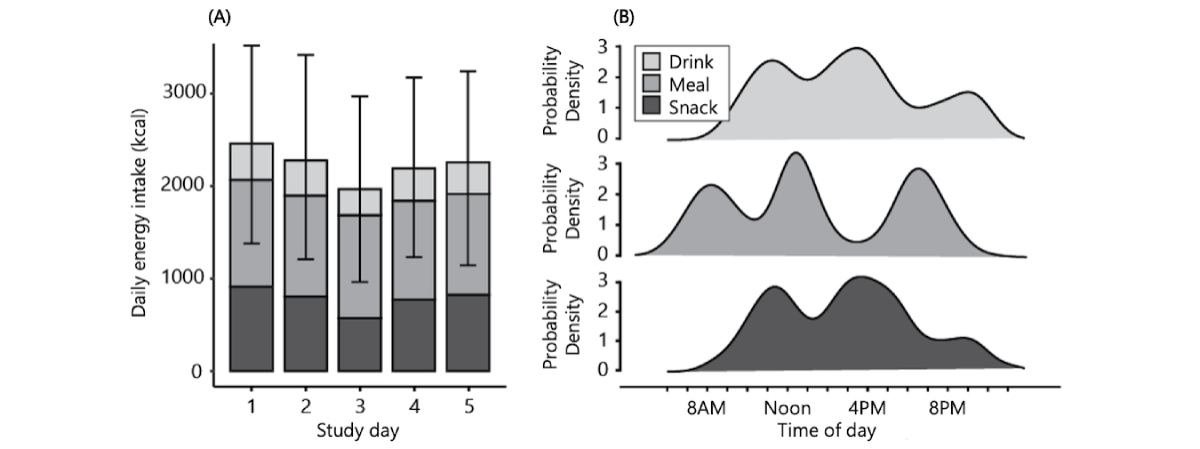
Daily consumption patterns. (A) Box plots showing the distribution of daily consumption (kcal) across drinks, meals, and snacks per study day. (B) Density plots illustrating the temporal distribution of all drinking, meal, and snacking events recorded across participants and measurement days.

During the study period, participants recorded 704 meals, and the SnackBox registered 2199 snacking events. This resulted in an average consumption of 2227 (SD 1078) kcal per day, with 769 (34.5%; SD 772) kcal from snacks and 351 (15.8%; SD 448) kcal from beverages. Figure 5A shows the distribution of snack and drink consumption across the 5 measurement days. The density plots in Figure 5B illustrate the temporal distribution of all eating events recorded across participants and measurement days; visually, elevated snack and drink consumption appears around 11 AM, 3:30 PM, and 9 PM. Additional details on consumption times for each food product are available in Multimedia Appendix 2.

### Case Study 1: Effect of Stress on Snacking Behavior

**Figure 6:**
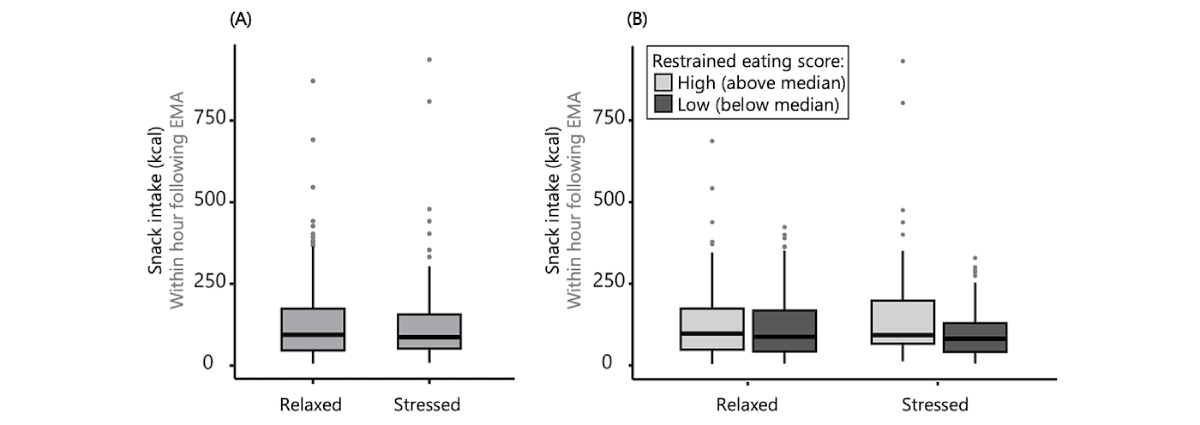
Impact of stress and eating restraint on post–ecological momentary assessment (EMA) snacking behavior. (A) Box plots comparing caloric snack intake during the hour following EMA responses for participants reporting relaxed (visual analog scale [VAS] score<50) vs stressed (VAS score>50) states. (B) Box plots displaying caloric snack intake following EMA responses, stratified by both stress level and restrained eating scores (Dutch Eating Behavior Questionnaire–restrained eating; high=above median; low=below median).

A total of 1455 EMA questionnaires were completed on measurement days, with participants completing an average of 6.6 (SD 4.8) questionnaires per day. Excluding neutral responses (VAS=50; n=192, 13.2% observations), 1263 (86.8%) responses were retained for analysis (Figure 4), of which 357 (28.3%) indicated stress (VAS>50) and 906 (71.7%) indicated relaxation (VAS<50). The average stress score across all nonneutral responses was 63.1 (SD 22.0). Stress levels did not significantly influence the likelihood of snack consumption in the hour following an EMA (*χ*^2^_1_=0.2; *P*=.69). Across all observations, snacks were consumed in 24% of cases (for snacks, *P*=.24). Among instances where snacking occurred within the hour following an EMA (n=302, 23.9% observations), total caloric intake from snacks was not significantly affected by stress levels (*χ*^2^_1_=0.6; *P*=.45; Figure 6).

Although Figure 6 suggests some variations in caloric intake between high and low restrained eaters when experiencing stress, no significant effect of restrained eating on snack consumption was found (*χ*^2^_1_=2.5; *P*=.11), nor was there a significant interaction effect between stress levels and restrained eating (*χ*^2^_1_=0.1; *P*=.75). These findings suggest that stress does not significantly influence the likelihood of snacking or caloric intake from snacks. Additionally, restrained eaters did not exhibit a greater propensity for stress-induced snacking. Similar analyses for sex and emotional eating yielded comparable results, which are available in the Multimedia Appendix 3.

### Case Study 2: Snacking in Relation to PA

**Figure 7:**
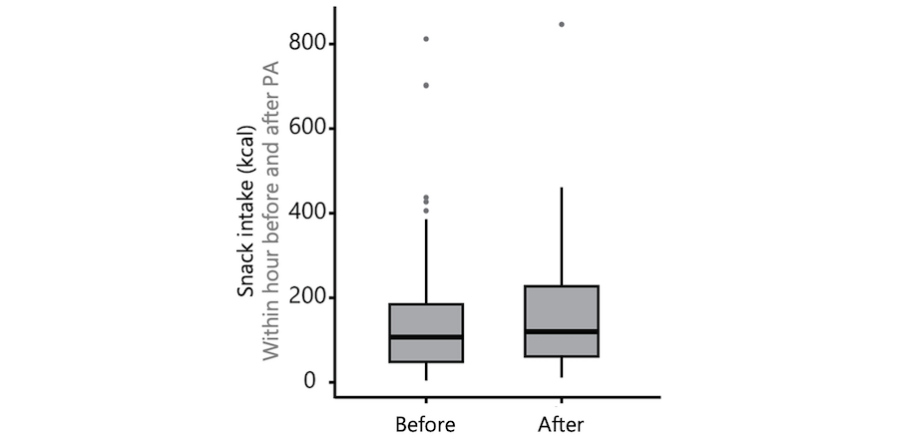
Box plots comparing caloric snack intake during 1-hour windows before vs after vigorous physical activity (PA).

Over the study period, 2933 hours of Chill+ data were collected. Seven hundred thirteen periods of vigorous PA were identified, corresponding to a mean of 4.3 (SD 3.4) periods per participant per measurement day (Figure 4). Daily energy intake from snacks was not significantly associated with mean daily METs (*P*=.74) or daily minutes of vigorous PA (*P*=.63), after accounting for participant variation and the number of days into the study. Snack data of 613 one-hour windows prior to and 625 one-hour windows after vigorous PA were available for analysis. In these hours, time relative to vigorous PA was associated with the likelihood of snacking (*χ*^2^_1_=12.9; *P*<.001). The probability of snacking prior to PA (for snack, *P*=.14; 95% CI 0.10-0.19) was higher compared with after PA (for snack, *P*=.07; 95% CI 0.05–0.11), corresponding to an odds ratio of 2.00 (95% CI 0.94-4.28; *P*<.001; CI back-transformed from the log-odds scale). Among windows in which snacking occurred (n=139, 11.2% observations), the total caloric consumption of snacks was not different between consumption prior to and following PA (*χ*^2^_1_=0.4; *P*=.55), as illustrated in Figure 7. This suggests that participants were more likely to snack before PA than after PA; however, while snacking, PA did not affect energy consumption.

## Discussion

### Principal Findings

In this study, we demonstrated the utility of the SnackBox as a technology probe for investigating snacking in relation to mood and physiological states in structured, seminaturalistic environments. Results from our case studies revealed no effect of perceived stress on snacking behaviors. However, snacking was less likely following vigorous PA compared with periods prior to such activity (odds ratio 2.00; SE 0.39; *P*<.001).

Our case study found no effect of perceived stress on snack occurrence or caloric intake, consistent with previous findings [32,46-48], but contrasting with results from a laboratory-based study [49], a daily-diary study [50], and a meta-analysis on stress-induced eating [19]. Hill et al [19] reported a small overall effect of stress on food intake, moderated by restrained eating traits. Although some variation was observed between high and low restrained eaters, these differences did not reach statistical significance (*P*=.11). The absence of an effect may be due to the exploratory nature of our analysis, which prioritized our primary research objective, and the sample’s low restrained eating scores, likely influenced by inclusion criteria excluding dietary restrictions. Stress-induced eating generally has a small effect size, with only 35% to 40% of individuals increasing intake under stress [19,51]. Although our sample size was not specifically powered to detect such small effects, studies with similar sample sizes (eg, van Strien et al [49]) have reported significant findings. Additionally, we did not account for snack “healthiness,” which could obscure stress-related eating patterns, as prior studies suggest stress increases intake of unhealthy foods and decreases intake of healthy foods [19]. Methodological limitations, such as potential biases from VAS scores set to default positions or the use of both negative and positive affect on one scale [52], also warrant adjustment in future studies.

Our second exploratory case study found that participants were more likely to snack before rather than after PA, consistent with studies on exercise-induced appetite suppression [44,53,54]. Oh and Taylor [33] demonstrated that moderate and vigorous exercise acutely reduce snack cravings and attentional bias toward food cues in a controlled setting, and King et al [44] reported delayed initiation of eating after intense exercise compared with a control group. King et al [44] reported no impact on total food intake—mirroring our finding of no link between snack caloric intake and vigorous PA, although we did not account for meal energy intake. While prior studies primarily focused on single bouts of aerobic or resistance exercise [33,34], our findings indicate similar food intake responses to periods of vigorous PA scattered throughout the day, as measured continuously via wearables. Appetite suppression is attributed to increases in anorexigenic hormone levels and decreases in orexigenic hormone levels, which normalize hours after exercise [53,54]. Further research is needed to confirm if short bouts of vigorous activity influence appetite through these underlying pathways in naturalistic settings. Additionally, we did not account for adiposity and habitual activity levels, which potentially modulate appetite regulation [34].

The EMA questionnaires used in this study represent a state-of-the-art method for capturing targeted data points during moments of interest, facilitating the investigation of snacking behaviors and their determinants, such as cravings [55] or food environments [56]. EMA prompts were sent both randomly and triggered upon snack retrieval from the SnackBox. However, future studies should prioritize random sampling, as triggered prompts may discourage participants from snacking due to the anticipated effort of completing an EMA questionnaire [28]. Moreover, triggered prompts lead to skewed sampling, with more snacks recorded before rather than in the hour following EMA completion. Future applications could explore continuous-time models for EMA data [57] to estimate mood fluctuations throughout the day, aligning them with precise snack consumption times recorded by the SnackBox. These models would also facilitate the analysis of potentially delayed effects of stress or negative emotions on eating using statistical methods with variable time lags [58]. Additionally, recent efforts to recognize emotions via physiological signals from wearables [59] could eventually bypass all self-reporting methods for studying emotional states in relation to snacking behaviors.

The temporal distribution of snacking events recorded by the SnackBox visually aligned with findings from Dutch national consumption surveys [1,60], with elevated consumption appearing around midmorning, midafternoon, and evening hours, though these patterns were not tested statistically. Participants in this study derived a greater proportion of their daily energy intake from snacks consumed between regular meals (1120/2227, 50.3% kcal) compared with the Dutch national average of 28% (582/2078 kcal) [1]. This discrepancy may be attributed to underreporting in national dietary surveys, which rely on self-reported methods known to underestimate snack consumption [22,23,28]. However, several aspects of our study design might also clarify this elevation and should be acknowledged as study limitations. The snack selection did not include fruits and vegetables due to their shorter shelf life, leading to a predominance of energy-dense options likely to result in higher energy intake [61]. The exclusion of individuals with dietary restrictions was necessitated by the fixed snack selection, which was designed to support the initial validation of the device [28], limiting the generalizability of findings to the broader population and warranting expanded snack offerings in future studies. Additionally, participants were instructed to consume at least one bite of a snack or sip of a beverage per measurement day to verify device functionality, which may have further inflated snacking frequency. Furthermore, as highlighted by participants in the study by de Gooijer et al [28], the placement of the SnackBox on participants’ desks may have amplified consumption due to the proximity effect [62]. These findings highlight the need for methodological refinements in future studies to minimize potential biases in snacking behavior. Possible adaptations include allowing participants to incorporate self-selected snacks and positioning the SnackBox in typical snack storage locations, such as cupboards or refrigerators, to better reflect habitual snacking patterns.

In various fields, there is a growing shift from self-administered to passive methodologies. Activity trackers are widely used to monitor PA [55] and sleep patterns [63], while GPS technology enables the tracking of reported locations [64]. In dietary assessments, most passive technologies rely on image-based methods [65,66], which, despite their potential, raise privacy concerns, pose challenges in portion size estimation, and still demand manual evaluation by dietitians or researchers. Other approaches, such as motion-based [67] and sound-based [68] detection of eating events, fail to capture information about the type or quantity of food consumed. The SnackBox has emerged as a promising tool for obtaining reliable dietary intake time-series data in real-world settings [28]. Combining such data with information on physiology, mood, or eating context in larger, diverse cohorts or longitudinal studies could deepen our understanding of snacking behavior dynamics. This knowledge could, for example, inform the development of personalized intervention programs [69] or predictive modeling for just-in-time adaptive interventions [70].

### Conclusions

In conclusion, the SnackBox demonstrated utility as a technology probe for studying snacking behavior in seminaturalistic settings. Perceived stress did not significantly influence snacking likelihood or caloric intake, while snacking was more likely before than after vigorous PA, consistent with exercise-induced appetite suppression. Integrating automated dietary data with concurrent physiological and contextual measures offers a promising approach for deepening our understanding of snacking behavior and informing personalized interventions and predictive models.

## Appendix

Multimedia Appendix 1 Available snack and drink options and their nutritional information.

Multimedia Appendix 2 Density plots illustrating the temporal distribution of drinks and snack consumption events throughout the day.

Multimedia Appendix 3 Impact of stress, emotional eating and sex on post–ecological momentary assessment (EMA) snacking behaviour. (A) Box plots comparing caloric snack intake during the hour following EMA responses for participants reporting relaxed (visual analog scale [VAS] score<50) vs stressed (VAS score>50) states stratified by emotional eating score. (B) Box plots displaying caloric snack intake following EMA responses, stratified by both stress level and sex. (Dutch Eating Behaviour Questionnaire–emotional eating; high=above median; low=below median).

## Abbreviations

DASS: Depression Anxiety Stress Scale
DEBQ: Dutch Eating Behavior Questionnaire
DEBQ-E: Dutch Eating Behavior Questionnaire–emotional eating
DEBQ-R: Dutch Eating Behavior Questionnaire–restrained eating
EMA: ecological momentary assessment
MET: metabolic equivalent of task
PA: physical activity
PSS: Perceived Stress Scale
RFID: radio-frequency identification
VAS: visual analog scale
