# Supplementary material for: Snacking Behaviors in Relation to Stress and Physical Activity Using Automated Dietary Assessment: Observational Study
Source: JMIR Form Res. 2026 Jul 23;10:e77046. doi: 10.2196/77046 (PMC13448199; doi:10.2196/77046)
Supplement: Supplementary file 1 [file formative_v10i1e77046_app1.docx]

**Supplemental Material A. Available snack and drink options and their nutritional information.**

| Snacks | Brand | Energy (kcal/100g) | Carbohydrates (g/100g) | Of which sugar (g/100g) | Fat (g/100g) | Of which saturated (g/100g) | Of which unsaturated (g/100g) | Protein (g/100g) | Fiber (g/100g) | Salt (g/100g) | URL |
| --- | --- | --- | --- | --- | --- | --- | --- | --- | --- | --- | --- |
| Winegums | Albert Heijn | 334 | 83 | 52 | 0.2 | 0.2 | 0 | 0.1 | 0 | 0.13 | [AH Winegums bestellen \| Albert Heijn](https://www.dutchexpatshop.com/en/chaudfontaine-fusion-lemon.html) |
| Salted peanuts | Albert Heijn | 609 | 9.6 | 5 | 50 | 6 | 43 | 26 | 8.5 | 0.7 | [AH Gezouten Pinda's bestellen \| Albert Heijn](https://www.jumbo.com/producten/aquarius-lemon-daily-hydration-500-ml-432250FLS) |
| Unsalted peanuts | Albert Heijn | 620 | 11 | 5.4 | 51 | 8 | 43 | 25 | 8.4 | 0 | [AH Ongezouten pinda's bestellen \| Albert Heijn](https://www.jumbo.com/producten/fanta-orange-zero-sugar-0,5-l-479104FLS) |
| Savoury biscuits | Sultana | 404 | 68 | 10 | 9.9 | 1.5 | - | 8.3 | 4.4 | 2.29 | [Sultana Crunchers tomaat & kaas bestellen \| Albert Heijn](https://www.jumbo.com/producten/fuzetea-green-tea-mango-chamomile-12-x-400-ml-432258PKL) |
| Rice snacks | Albert Heijn | 400 | 77 | 7.7 | 6.2 | 1.4 | 4.8 | 7.7 | 2.8 | 1.33 | [AH Japanse mix bestellen \| Albert Heijn](https://www.ah.nl/producten/product/wi421650/celebrations-assortiments-mix) |
| Raisins | Albert Heijn | 344 | 80 | 59 | 0.5 | 0.1 | 0 | 3 | 3.5 | 0 | [AH Rozijnen bestellen \| Albert Heijn](https://www.jumbo.com/producten/minute-maid-apple-330-ml-647638TRL) |
| Unsalted nuts | Albert Heijn | 668 | 6.1 | 3 | 61 | 6.9 | 53 | 20 | 7.1 | 0 | [AH Ongezouten notenmix bestellen \| Albert Heijn](https://www.ah.nl/producten/product/wi467592/ah-gezouten-pinda-s) |
| Muesli bars | Albert Heijn | 344 | 51 | 13 | 7.6 | 0.9 | 6.5 | 7 | 22 | 0.1 | [AH Mueslireep naturel bestellen \| Albert Heijn](https://www.ah.nl/producten/product/wi467588/ah-ongezouten-pinda-s) |
| M&M’s (crispy) | M&M’s | 485 | 68 | 57 | 21 | 14 | - | 4.3 | - | 0.35 | [M&M'S Crispy melk chocolade bestellen \| Albert Heijn](https://www.ah.nl/producten/product/wi588154/sultana-crunchers-tomaat-en-kaas) |
| M&M’s (chocolate) | M&M’s | 481 | 70 | 65 | 19 | 12 | - | 5.2 | - | 0.2 | [M&M'S Melk chocolade bestellen \| Albert Heijn](https://www.ah.nl/producten/product/wi468279/ah-japanse-mix) |
| M&M’s (peanut) | M&M’s | 526 | 59 | 52 | 27 | 11 | - | 10 | - | 0.15 | [M&M'S Pinda melk chocolade bestellen \| Albert Heijn](https://www.ah.nl/producten/product/wi584104/ah-winegums) |
| Dried apricots | Albert Heijn | 770 | 37 | 37 | 0.6 | 0 | 0.6 | 4 | 6.3 | 0.03 | [AH Gewelde gedroogde abrikozen bestellen \| Albert Heijn](https://www.jumbo.com/producten/sprite-zero-sugar-lemon-lime-500-ml-553404FLS) |
| Cocktail nuts | Albert Heijn | 531 | 42 | 7.6 | 33 | 4.8 | 28 | 14 | 5.1 | 1.68 | [AH Borrelnoten cocktail bestellen \| Albert Heijn](https://www.ah.nl/producten/product/wi407177/ah-mueslireep-naturel) |
| Cheese biscuits | Albert Heijn | 574 | 46 | 2.5 | 37 | 23 | 13 | 14 | 0.3 | 2.25 | [AH Kaaskoekjes met roomboter bestellen \| Albert Heijn](https://www.ah.nl/producten/product/wi569837/m-en-m-s-crispy-melk-chocolade) |
| Celebrations | Mars, Incorporated | 495 | 62 | 55 | 24 | 14 | - | 5.6 | - | 0.42 | [Celebrations Assortiments mix bestellen \| Albert Heijn](https://www.ah.nl/producten/product/wi164746/ah-rozijnen) |

| Drinks | Brand | Energy (kcal/100ml) | Carbohydrates (g/100ml) | Of which sugar (g/100ml) | Fat (g/100ml) | Of which saturated (g/100ml) | Of which unsaturated (g/100ml) | Protein (g/100ml) | Fiber (g/100ml) | Salt (g/100ml) | URL |
| --- | --- | --- | --- | --- | --- | --- | --- | --- | --- | --- | --- |
| Sports drink | Aquarius | 19 | 4.4 | 4.4 | 0 | 0 | - | 0 | - | 0.05 | [Aquarius Lemon Daily Hydration 500 ml bestellen? \| Jumbo](https://www.ah.nl/producten/product/wi388601/ah-gewelde-gedroogde-abrikozen) |
| Orange soda (light) | Fanta | 3 | 0.4 | 0.4 | 0 | 0 | - | 0 | - | 0.01 | [Fanta orange zero sugar 0,5 L bestellen? \| Jumbo](https://www.jumbo.com/producten/fuze-tea-infused-iced-green-tea-12-x-400-ml-557604PKL?channable=02a13d696400343739313034464c5386&utm_campaign=alwayson&utm_medium=cpc&utm_source=google&ju_subth=b2c&utm_content=b2c_NL_ecommerce_shopping-non-brand_segmented&utm_id=21474612135_196255346314_479104FLS&gad_source=1&gad_campaignid=21474612135&gclid=Cj0KCQjwgr_NBhDFARIsAHiUWr6_JJRjHOtoUFOEybhzGSzcB5uEKe6UqliMtQ7Aa3OlA4m8BJ--gZsaAjTWEALw_wcB) |
| Orange soda | Fanta | 19 | 4.4 | 4.4 | 0 | 0 | - | 0 | - | 0.02 | [Fanta Orange PET Fles 500 ml bestellen? \| Jumbo](https://www.ah.nl/producten/product/wi221560/m-en-m-s-melk-chocolade) |
| Orange juice | Minute Maid | 43 | 10 | 10 | 0 | 0 | - | 0 | - | 0.01 | [Minute Maid Orange Nectar 200 ml bestellen? \| Jumbo](https://www.ah.nl/producten/product/wi228068/m-en-m-s-pinda-melk-chocolade) ^1^ |
| Lemon water | Chaudfontaine | 0.4 | 0 | 0 | 0 | 0 | - | 0 | - | 0 | [Chaudfontaine Fusion lemon Order Online \| Worldwide Delivery](https://www.ah.nl/producten/product/wi426102/ah-ongezouten-notenmix?srsltid=AfmBOoqwre3PHIieBDFVxp_ugNighxANEQwBrQnNEGc5cNzvJEudM6Kl) |
| Lemon soda (light) | Sprite | 1 | 0 | 0 | 0 | 0 | - | 0 | - | 0.04 | [Sprite Zero Sugar Lemon-Lime 500 ml bestellen? \| Jumbo](https://www.ah.nl/producten/product/wi578191/ah-borrelnoten-cocktail) |
| Iced green tea | Fuze Tea | 19 | 4.4 | 4.4 | 0 | 0 | - | 0 | - | 0.05 | [Fuze Tea Infused Iced Green Tea 12 x 400 ml bestellen? \| Jumbo](https://www.ah.nl/producten/product/wi129543/ah-kaaskoekjes-met-roomboter) |
| Iced black tea | Fuze Tea | 19 | 4.3 | 4.3 | 0 | 0 | - | 0 | - | 0.03 | [FuzeTea Green Tea Mango Chamomile 12 x 400 ml bestellen? \| Jumbo](https://www.jumbo.com/producten/fanta-orange-pet-fles-500-ml-434372FLS) |
| Apple juice | Minute Maid | 45 | 10.7 | 10.1 | 0 | 0 | - | 0.1 | - | 0.1 | [Minute Maid Apple 330 ml bestellen? \| Jumbo](https://www.jumbo.com/producten/minute-maid-orange-nectar-200-ml-580854KRT) ^1^ |

^1^Available in resealable PET bottles (330 ml); product has since been discontinued.
